# Longitudinal cognitive screening study in community-dwelling individuals

**DOI:** 10.1590/S1980-57642010DN40300010

**Published:** 2010

**Authors:** Carolina P.M. Pereira, Florindo Stella, Salma S.S. Hernandez, Larissa P. Andrade, Camila V.L. Texeira, Sebastião Gobbi

**Affiliations:** 1 Biologist, Biosciences Institute, Universidade Estadual Paulista (UNESP), Campus of Rio Claro, SP, Brazil.; 2 MD, PhD, Aging and Physical Activity Laboratory (LAFE), Biosciences Institute, Universidade Estadual Paulista (UNESP), Campus of Rio Claro, SP, Brazil and Geriatric Psychiatry Clinic, Medical School, State University of Campinas (UNICAMP), Campinas SP, Brazil.; 3 MD Research on Motricity Sciences, Aging and Physical Activity Laboratory (LAFE), Biosciences Institute, Universidade Estadual Paulista (UNESP), Campus of Rio Claro, SP, Brazil.; 4 MD, PhD, Coordinator of the Aging and Physical Activity Laboratory (LAFE), Biosciences Institute, Universidade Estadual Paulista (UNESP), Campus of Rio Claro, SP, Brazil.

**Keywords:** longitudinal study, cognitive screening, dementia

## Abstract

Cognitive changes in normal aging can be similar to the alterations that take place in the initial stages of a dementia process. Longitudinal studies can provide a better understanding of this progression.

**Objectives:**

To evaluate the cognitive and functional evolution of community-dwelling individuals without dementia through a three-year longitudinal study.

**Methods:**

168 individuals were evaluated in 2006. Three years later in 2009, 73 of these subjects were reevaluated as regards cognition and functionality using the Mini Mental State Examination (MMSE), Brief Cognitive Battery (BCB) and the Pfeffer Functional Activities Questionnaire. The statistical analysis included descriptive measurements, the Wilcoxon’s test for intra-group comparison, and the Spearman’s correlation coefficient test for comparing cognitive and functionality scores.

**Results:**

After three years, the Wilcoxon’s test showed a discreet yet significant cognitive decline (MMSE: –0.7 points; p=0.02; Z= –2.29; and global score on the BCB: +3.6 points; p=0.02; Z= –2.29), in addition to functional decline (Pfeffer: +0.7 points; p= 0.001; Z= –3.38).

**Conclusions:**

After three years of follow-up we observed a discreet yet significant functional and cognitive decline in the subjects. Longitudinal cognitive screening represents an important strategy in the early identification of changes from normal conditions to a dementia process.

## Abstract (pt)

Mudanças cognitivas no envelhecimento normal podem se assemelhar às alterações nos períodos iniciais de um processo demencial. Estudos longitudinais possibilitam um entendimento melhor desta progressão.

**Objetivos:**

Avaliar, em um estudo longitudinal de três anos, a evolução cognitiva e funcional de sujeitos sem demência da comunidade.

**Métodos:**

Após três anos de uma primeira avaliação de 168 sujeitos, em 2006, 73 sujeitos foram reavaliados em 2009 quanto à cognição e funcionalidade, através do Mini-Exame do Estado Mental (Mini-Mental), Bateria Breve de Rastreio Cognitivo (BBRC) e Questionário de Atividades Instrumentais (Pfeffer). A análise estatística constou de medidas descritivas, teste de Wilcoxon para comparação intragrupo e coeficiente de correlação de Spearman para comparação entre escores cognitivos e de funcionalidade.

**Resultados:**

Após três anos, o teste de Wilcoxon evidenciou declínio cognitivo discreto, porém significativo (Mini Mental: –0.7 pontos; p=0.02; Z= –2.29; e escore global da BBRC: +3.6 pontos; p=0.02; Z= –2.29), além de declínio funcional (Peffer: +0.7 pontos; p= 0.001; Z= –3,38).

**Conclusões:**

Após três anos de seguimento, observou-se discreto, porém significativo, declínio cognitivo e funcional dos sujeitos. A BBRC e o Mini-Mental mostraram-se eficazes para o screening cognitivo. O seguimento cognitivo longitudinal representa uma estratégia importante para a identificação precoce de possível evolução de uma condição de normalidade para um processo demencial.

Over the last few decades there has been much discussion on the threshold between normal and altered cognition. The discussion seeks to identify the presence of possible determining or related factors to the onset of a dementia condition. According to Damasceno,^1^ the normal aging of the brain can present a series of mental alterations similar to those in an early stage of dementia when symptoms are still not clear. This similarity can make it difficult to distinguish between cognitive alterations typical of aging and those of Alzheimer’s disease. In spite of the consensus regarding neuropsychological, neuropathological and neuroimaging criteria to distinguish between normal and pathological states, this differential diagnosis poses a major challenge in clinical practice.^1^ Longitudinal studies provide relevant information concerning cognitive alterations in normal aging, mild cognitive impairment, and the early stage of dementia.^2,3^ Among cognitively preserved individuals, the mental processes and functionality remain devoid of clinically relevant alterations, for instance in episodic memory, semantic memory, working memory, visuospatial ability, and perceptual speed.^2^ In addition, the category called cognitive impairment non-dementia includes cases which not fulfill the criteria for dementia. In these cases, mild and stable alterations can be observed in mental processes such as learning, reasoning, language, executive functions, attention, and concentration, akin to the changes seen in mild cognitive impairment or in the early stage of dementia.^1-3^

The early identification of the risks for dementia, mainly Alzheimer’s type, is of utmost importance,^4^ since the therapeutic intervention in the initial stages can stabilize cognitive status and provide functional and behavioral improvement, at least temporarily.^5^ Additionally, early intervention tends to reduce the emotional suffering of patients and their caregivers and family members, as well as contributes to the administration of the general conditions of their personal and family lives.^6,7^ In this sense, early detection can prolong the autonomy of the subject and increase the chances of delaying the demential evolution that results from the neurodegenerative process.^7^

Given that the cognitive alterations typical of aging are not always easy to distinguish from the changes seen in the early stages of dementia, longitudinal studies are a sensitive means of detecting the progression from cognitive decline to a possible condition of dementia, particularly in individuals with better performance on neuropsychological tests.^8,9^

Neuropsychological evaluation represents a necessary strategy to allow discrimination among normal cognition, mild cognitive impairment and the initial stage of Alzheimer’s.^10^ It is an integral part of the strategies used to diagnose cognitive decline, being clinically relevant and helpful in distinguishing among the various types of dementia evolution.^11,12^ Although the diagnosis of a dementia condition should be established according to clinical criteria adopted internationally,^13^ the evaluation of an individual with suspected dementia requires confirmation and assessment of cognition by means of neuropsychological evaluation. This evaluation should take into consideration factors that may interfere in the performance of individuals on these tests, such as the typical educational heterogeneity observed in the Brazilian population.^11^ However, one should bear in mind that the neuropsychological evaluation consists of a complex strategy that takes a relatively long time to carry out. On the other hand, population studies that aim to perform cognitive screening suggest the use of instruments that are easy to apply and demand a relatively short time.^14^

In Brazil, the Brief Cognitive Battery^15,16^ represents an effective cognitive screening strategy for the heterogeneous groups of individuals which are characteristic of the Brazilian population. This instrument has been used to identify cognitive alterations, particularly those related to memory, in individuals with suspected dementia.^15,16^

In our area, previous investigations based on the follow-up of the cognitive profile of individuals over time are scant. Therefore, the present investigation consisted of a three-year longitudinal study that aimed to evaluate the cognitive and functional evolution of community-dwelling individuals without dementia.

## Methods

### Subjects

**Figure 1:**
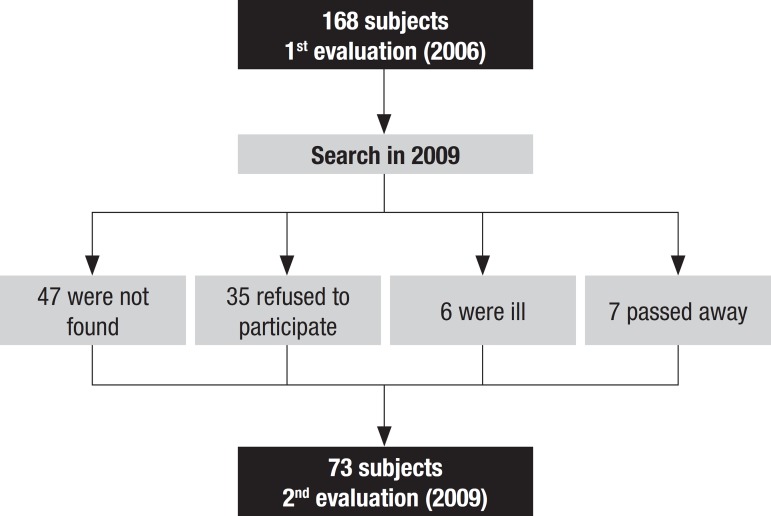
Flowchart of participants, depicting the 1st and 2nd evaluation of the study.

The study was a longitudinal investigation spanning three years from 2006 to 2009. The evaluations, carried out at two time points, were applied by trained raters. Initially in 2006 (1^st^ evaluation), 168 individuals without dementia from a community-dwelling in Rio Claro, São Paulo State, Brazil, (103 females and 65 males) were investigated. The subjects included were participating in a program of physical activity aimed at the elderly (Programa de Atividade Física para Terceira Idade [Program of Physical Activity for the Third Age] - PROFIT, UNESP, campus of Rio Claro). Over the period, the cognitive performance and activities of daily living of participants was assessed. In 2009 (2^nd^ evaluation), three years after the first assessment, the 73 subjects (48 females and 25 males) who remained in the study were reassessed. Thus, there was a sample loss of 95 subjects with 35 individuals refusing to participate mainly owing to sickness, 6 were ill, 7 passed away, and 47 could not be found by the researchers. Figure 1 shows the flowchart of the participants.

The Research Ethics Committee of the Institute of Biosciences, UNESP - Universidade Estadual Paulista, campus Rio Claro, SP, approved the study. All the participants signed a written informed consent form.

### Assessments

As part of the assessment, the authors devised and applied a Socio-demographic Questionnaire with questions on personal data, educational level, pathologies and medication prescriptions. The subjects’ cognitive profile was assessed by the Mini-Mental State Examination (MMSE)^17,18^ and the Brief Cognitive Battery (BCB),^15,16^ as well as by the Verbal Fluency Test^19^ and the Clock Drawing Test.^20^ The values of the items in the BCB were converted into global scores according to the mathematical formula established by Nitrini et al.^21^ The BCB can be considered in two ways: according to the individual items or to the global score. Global scoring entails the application of the mathematical formula mentioned above which reverses the way values from individual items are construed. As explained by Nitrini et al.,^21^ this calculation procedure includes four items: three items from the BCB - learning, delayed recall and verbal fluency scores - and years of schooling. Thus, while high scores from individual items indicate better performance, application of the mathematical formula means that a high global score indicates low cognitive performance. The Pfeffer Functional Activities Questionnaire was used to identify the level of functionality of the subjects required in the instrumental activities of their daily routine.^22^

### Statistical analysis

The statistical analysis initially comprised descriptive measures of the data (mean and standard deviation) and assessment of their distribution using the Shapiro Wilk’s test. Since the data did not present a normal distribution, the Wilcoxon’s test was applied for intra-group comparison. Moreover, Spearman’s correlation coefficient was applied to identify possible correlations between the scores in the cognitive and activities of daily living tests. The level of significance was set at 5%.

## Results

From the original sample of 168 participants with mean age of 69.9 years (±7.5 years; 57 to 90 years) and formal education of 6.5 years (±5.5 years), 73 remained in the second study three years later. These individuals had a mean age of 68.1 years (±7.0 years; 59 to 90 years) and 7.2 years (±6.2 years) of formal education. Therefore, 95 individuals were not involved in the second study because of their clinical condition, death or refusals as outlined in methodological procedures. Interestingly, mean age was lower and educational level higher among individuals from the second study compared with that of participants from the first study. This occurred due to the higher concentration of older age and higher educational level of the individuals not involved in the second study.

Based on the data from the 2006 and 2009 assessments, the Wilcoxon test detected that the subjects presented a mild yet significant cognitive decline, according to the MMSE score and global score on the BCB. The MMSE score fell by 0.7 points (p=0.02; Z= –2.29) whereas the BCB total score increased by 3.6 points (p=0.02; Z= –2.29). As explained in the methodological procedures, the increase in the global score of the BCB indicates a cognitive decline (the opposite of when items are considered individually). The change resulted mainly from lower scores on the Verbal Fluency Test (–2.3 words) which was also significant on the Wilcoxon’s test (p= 0.001; Z= –4.13), and whose values are included in the global score of the battery. An increase in the score for the Pfeffer Functional Activities Questionnaire (+0.7 points) was also detected, a value also significant on the Wilcoxon’s test (p= 0.001; Z= –3.38). The increase in the scores of this instrument also represents poorer functioning.

No significant differences were detected between the two evaluations in the items of the BCB considered individually (identification, incidental memory, immediate memory, learning memory, 5-minute memory, and recognition), although a trend toward decline in the item *incidental memory* (p=0.06) was observed. No differences were detected between values in the evaluations of the Clock Drawing Test. Table 1 summarizes the results of the tests applied in the two evaluations with a three-year interval.

**Table 1:** Comparison between 1^st^ evaluation and 2^nd^ evaluation three years later of the 73 subjects, according to the Wilcoxon's test.

Instruments	1^st^ Evaluation (Mean±standard deviation)	2^nd^ Evaluation (Mean±standard deviation)	p
Mini-Mental State Examination	27.7±2.1	27.0±2.6*	0.02
BCB Identification Incidental Memory Immediate Memory Learning Memory 5-minute Memory Recognition Verbal Fluency	9.9±0.2 5.8±0.1 7.8±1.3 8.3±1.4 8.2±1.5 9.7±0.6 17.1±4.7	9.9±0.1 6.2±1.4 7.7±1.3 8.0±1.7 8.0±1.7 9.7±0.8 14.8±4.3*	0.56 0.06 0.51 0.24 0.37 0.78 0.01
BCB (global score)	8,5±15,1	12,1±21,0*	0.02
Clock Drawing Test	8.1±1.9	7.8±1.7	0.11
Pfeffer's Questionnaire	0.1±0.6	0.8±1.5*	0.01

* Significant difference between 1^st^ and 2^nd^ evaluation. BCB: Brief Cognitive Battery.

Based on Spearman’s coefficient, although in the second assessment it was not possible to establish correlations among results from Mini-Mental State Exam, BCB, and Pfeffer’s Questionnaire, a fortuitous association between the BCB and Mini-Mental State Exam (0.2; p<0.05) could be considered, suggesting that both instruments indicate cognitive decline. This fortuitous association was also evident between the BCB and Pfeffer’s Questionnaire (0.2; p<0.05), showing that it is plausible to deduce that cognitive decline contributes to functional impairment.

## Discussion

The present study showed that the community-dwelling individuals without dementia had a discreet yet relevant cognitive decline over the three-year study interval. This decline was registered by the Mini-Mental (reduction of 0.7 points) as well as the BCB (increase of 3.6 points in the global score), as measured by the Wilcoxon’s test. Unlike the items considered separately, an increase in global score of this battery represents cognitive decline. The score on Pfeffer’s Questionnaire also showed a slight rise, which indicates a slight worsening in the performance of activities of daily living. The cognitive decline detected by the BCB was due to lower performance on the Verbal Fluency Test considered in the global score of the battery. In terms of Verbal Fluency, the subjects presented a decline from 17.1 to 14.8 between the 1^st^ and the 2^nd^ evaluations, with a significant difference between the two assessments (p=0.01). This test allows researchers to evaluate semantic memory and generation of words within the semantic category established.^23^ Gomez and White^24^ observed that verbal fluency associated with a semantic category contributes to the discrimination between cognitively healthy elderly individuals and those in the initial phase of Alzheimer’s disease. In the present study, no differences were detected between the 1^st^ and 2^nd^ evaluations on the BCB except in incidental memory, in which a trend toward a decline was observed (p=0.06). This trend may be partially explained by the procedure itself whereby in the incidental recall assessment, the subject is first requested to name each figure presented to them and shortly after is asked to remember each drawing presented by the researcher. This procedure commonly takes the individual by surprise as they are not previously ‘prepared’ to recall the figures, and may lack attention to answer this particular task.

Normal old adults are expected to remain cognitively stable with preserved functional capacities for a long period. Longitudinal studies have not definitively established how many points a normal elderly person loses per year. A review carried out by Gauthier et al.^28^ identified several controversies concerning the measurement of cognitive and functional decline in patients with dementia. In this context, patients with dementia classified into a slow course of disease can lose 0 to 1.9 points on the MMSE per year.^28^ In comparison to baseline, our subjects lost 0.7 points when assessed three years later. This score may be considered an expected performance for normal cognitive functioning. However, further periodical follow up of these individuals represents an appropriate strategy since they could be at risk for conversion to dementia.

Spearman’s correlation coefficient pointed to an association between the Mini-Mental State Exam and the BCB, as well as between the BCB and Pfeffer’s Questionnaire, but not between the MMSE and Pfeffer’s Questionnaire. Over time, the BCB seems to be more sensitive than the MMSE in detecting the association between functionality and cognitive alterations among normal individuals without dementia. The relationship between the performance of daily living activities and the generation of words required in the performance of the verbal fluency tests was also observed in other studies on memory associated with language.^25,26^

Regarding the age and level of education of the group that remained in the study three years later, it is important to consider that this group had a lower mean age and slightly higher educational level than the original sample, despite the fact the second assessment was applied only three years later. This occurred because the individuals who did not take part in the second study were older and had less schooling overall. As shown in Figure 1, the exclusion of these subjects was due to illnesses that prevented them from participating in the study, and to deaths or refusals. In addition, other subjects were not found by the researchers. It is plausible to deduce that individuals who remained in the second study were probably more cognitively preserved compared to those included in the original sample.

The BCB is less influenced by educational level than the MMSE^16,27^ and ensures high accuracy in the identification of cognitive alterations in populations with heterogeneous educational level.^21^ Even though the correlations indicated that the cognitive decline was associated with lower performance in the activities of daily living assessed by the Pfeffer’s Questionnaire, the subjects in the study retained these abilities to a sufficiently high level to avoid diagnosis of dementia. Nevertheless, the risk of progression to this diagnosis should be taken into consideration.

Unlike our study involving individuals without dementia in which we observed a mean decline of 0.7 points on the MMSE over three years, other investigations have shown a considerably more aggressive course of decline in patients with Alzheimer’s disease. In a review of the literature on the progression of dementia in Alzheimer’s disease, Gauthier et al.^28^ found a mean cognitive decline of two to three points per year on the Mini-Mental State Exam, whereas some investigations detected a loss of up to five points a year on the MMSE test.

The subjects in this study did not present decline in the scores of the Clock Drawing Test, which suggests the preservation of executive functions. This instrument has been largely used to identify the dysexecutive syndrome present in several neurodegenerative conditions and whose main symptoms are related to functional disorganization in frontal cortical areas, such as dementia in Parkinson’s disease, Lewy Body Dementia and frontal-temporal dementia. The test also aids in the differential diagnosis between those pathologies and Alzheimer’s disease.^29^ The cognitive decline of the subjects in the present investigation was due to the alterations in the domain of semantic memory, used to generate words in the Test of Verbal Fluency, rather than to executive dysfunctions. In other words, the impairment in semantic memory occurs earlier than do alterations in the executive functions in healthy individuals, and may be a sign of possible early impairment of the temporal lobe.^30^ In addition, semantic memory is probably related to word generation within a particular category, and this interaction could contribute to help discriminate between cognitively preserved individuals and those in the early phase of Alzheimer’s disease.^23,24^ Persistent decline in several cognitive processes including semantic memory, is most likely associated with development of dementia.^1,11,24,30^

In the present study, statistical analysis showed verbal fluency to be the most impaired cognitive function comparing baseline and final assessments. The reasons explaining this peculiarity remain unclear. Reduction in mental processing speed could be a plausible hypothesis for clarifying this peculiarity, although the definitive causes are not known because we did not measure the speed of cognitive functions. Nevertheless, Bennet et al.^2^ suggested that processing speed is an important resource for cognitive activity.

The factors that might contribute to the maintenance of cognitive and functional capacities of individuals without dementia are an intriguing issue.

During their lifetime, an individual goes through discreet cognitive alterations inherent to normal aging that do not significantly interfere in their functional capacities. One of the factors that might contribute to the preservation of this stability has its root in “cognitive reserve”, a concept suggested some decades ago. This reserve is built throughout an individual’s life history, being made up of the intellectual resources collected during life experiences in the context of social interactions, education, participation in cognitively stimulating activities and involvement in socio-occupational activities that demand a certain level of complexity of intellectual exercise.^31^ In addition to the capacity for an engaging lifestyle, intellectual ability and educational level exerts a positive role in maintaining previous levels of functioning during healthy aging, and in this way, high educational levels contribute to constructing the cognitive reserve. According to Baldivia et al.,^31^ the effects of education on cognition construction, manifested by neuropsychological performance, are not linear. These authors emphasize that differences are more marked when illiterate individuals are compared to others with three years of formal education, while this difference is less prominent when individuals with higher levels of schooling are compared.^31^ Clearly, it is important to consider the quality of schooling and the kind of lifestyle engaged that individuals have experienced for a long period.

The “brain reserve” in turn, is the set of neurobiological resources that result from the brain structure and systems underlying intellectual activities.^32,33^ Enhanced understanding of lifestyle engaging in environmental enrichment may provide enlightenment into the mechanisms of brain and cognitive reserve and lead to new strategic approaches to improve endogenous brain reserve, maintain cognitive healthy condition, and to delay onset of neurodegenerative or neuropsychiatric manifestations.^31-33^

It is also assumed that brain capacity and cognitive reserve play a role of partial “protection” in the presence of neurodegenerative processes against the early triggering of a dementia condition, delaying the onset of cognitive and functional decline present in Alzheimer’s disease.^31,33^ The measurement of this reserve is complex since, in developing countries like Brazil, the variability of cognitive expression is heterogeneous, partially determined by the educational level and socio-economic conditions of the individual.^34^

Therefore, the early identification of the progression of the risk for Alzheimer’s is of utmost importance, since there are no known interventions that can block the neurodegenerative process at present.^35,36^ In this context, the assessment of the cognitive profile of community-dwelling elderly individuals by means of screening tests helps in the recognition of pre-dementia conditions and dementia in its early stage.^5^ Longitudinal studies allow researchers to estimate the speed of progression of the cognitive and functional decline in individuals at risk for Alzheimer’s. Conversion to Alzheimer’s depends, simultaneously, on the neurodegenerative process and the degree of resilience, both of which can reduce the aggressiveness of the disease.^28^ Resilience partially depends on the cognitive and brain reserves.^32,32^

On the other hand, a persistent decline in memory or other cognitive functions can suggest the onset of dementia. Accordingly, mild cognitive impairment but not yet demential, poses an important threat of progression to Alzheimer’s disease.^38^

Another relevant issue regards non-pharmacological strategies in the preservation of the cognitive and brain reserves. Cognitive training of the elderly with cognitive impairment has proved effective.^37^ Benefits of this kind of training for Alzheimer’s patients^38^ were also observed in cognitive rehabilitation strategies.^38-41^

Moreover, other experiences including the practice of aerobic exercise, contribute to the maintenance of these abilities, even in individuals at risk for dementia.^42^ Out of the 73 subjects of this study, only 21 (28.7%) followed a regular program of physical activity. However, several studies highlight the practice of physical exercise, preferably aerobic, as a relevant resource for cognitive and functional maintenance. Ruscheweyh et al.,^43^ in a recent controlled study, showed that a six-month program of physical exercise improved the episodic memory of healthy elderly. In a retrospective study, Floel et al.^44^ observed that elderly individuals who regularly practiced physical exercises such as walking, cycling, swimming or tennis, also showed better memory performance compared with sedentary elderly.

Benefits of this type would result from the activation of the cognitive and brain reserves, and be associated with the practice of intellectual activity and physical exercise.^32^ Several studies in animal models and humans have shown that cognitive and functional activities have a neurobiological support characterized by the implementation of brain neuroplasticity. In this event, there is the action of several biomarkers, mainly the brain-derived neurotrophic factor and the factor of vascular growth, in addition to phenomena such as synaptogenesis, neurogenesis, release of neurotransmitters, and other related mechanisms.^43-49^ Neurogenesis induced by cognitive activity and physical exercise has been demonstrated in the subgranular zone of the dentate gyrus of the hippocampus, and greatly depends on the activation of neurotrophins, especially on the brain-derived neurotrophic factor.^43,44,49,50^ These findings suggest physical exercise and cognitive training stimulate brain plasticity, activate brain reserve and contribute to the implementation of cognitive reserve.^32^

In conclusion, after three years of follow-up we detected discreet yet significant cognitive and functional decline in the subjects studied. The BCB and the MMSE proved effective for the cognitive screening of this longitudinal study. Longitudinal assessment represents an important strategy for following up the cognitive profile of individuals without dementia and for identifying risk for progression from normal conditions to a dementia process.

The impact of psychosocial and neurobiological factors in the protection against cognitive and functional decline over time, as well as of non-pharmacological interventions such as cognitive training and physical exercise, represent a challenge for future studies. These interventions could contribute to the implementation of the neuroplasticity process increasing both cognitive and brain reserve.
